# Regulation of Heparanase in Diabetes-Associated Pancreatic Carcinoma

**DOI:** 10.3389/fonc.2019.01405

**Published:** 2019-12-10

**Authors:** Rachel Goldberg, Amichay Meirovitz, Alexia Abecassis, Esther Hermano, Ariel M. Rubinstein, Daniela Nahmias, Albert Grinshpun, Tamar Peretz, Michael Elkin

**Affiliations:** Sharett Oncology Institute, Hadassah-Hebrew University Medical Center, Jerusalem, Israel

**Keywords:** heparanase, pancreatic carcinoma, diabetes, hyperglycemia, extracellular matrix

## Abstract

While at least six types of cancer have been associated with diabetes, pancreatic ductal adenocarcinoma (PDAC) and diabetes exhibit a unique bidirectional relationship. Recent reports indicate that majority of PDAC patients display hyperglycemia, and ~50% have concurrent diabetes. In turn, hyperglycemic/diabetic state in PDAC patients fosters enhanced growth and dissemination of the tumor. Heparanase enzyme (the sole mammalian endoglycosidase degrading glycosaminoglycan heparan sulfate) is tightly implicated in PDAC progression, aggressiveness, and therapy resistance. Overexpression of heparanase is a characteristic feature of PDAC, correlating with poor prognosis. However, given the lack of heparanase expression in normal pancreatic tissue, the regulatory mechanisms responsible for induction of the enzyme in PDAC have remained largely unknown. Previously reported inducibility of heparanase gene by diabetic milieu components in several non-cancerous cell types prompted us to hypothesize that in the setting of diabetes-associated PDAC, hyperglycemic state may induce heparanase overexpression. Here, utilizing a mouse model of diet-induced metabolic syndrome/diabetes, we found accelerated PDAC progression in hyperglycemic mice, occurring along with induction of heparanase in PDAC. *In vitro*, we demonstrated that advanced glycation end-products (AGE), which are largely thought as oxidative derivatives resulting from chronic hyperglycemia, and the receptor for AGE (RAGE) are responsible for heparanase induction in PDAC cells. These findings underscore the new mechanism underlying preferential expression of heparanase in pancreatic cancer. Moreover, taken together with the well-established causal role of the enzyme in PDAC progression, our findings indicate that heparanase may sustain (at least in part) reciprocal causality between diabetes and pancreatic tumorigenesis.

## Introduction

Pancreatic ductal adenocarcinoma (PDAC) is one of the deadliest forms of malignancy and expected to become the second-leading cause of cancer-related death in the United States by 2030 (1). Dysregulation of glucose metabolism occurs in majority of PDAC patients: at PDAC diagnosis up to 85% of subjects have hyperglycemia and ~50% have diabetes (2–4). In a subset of PDAC patients diabetes occurs as early as 1–3 years before a detection of PDAC and is regarded as “new onset diabetes” (2–5). Long-standing type 2 diabetes also acts as a risk factor for pancreatic cancer (6, 7). Thus, elevation of glucose is a common phenomenon in PDAC (2–4). Additionally, positive association was reported between PDAC and insulin resistance/hyperinsulinemia (3, 8, 9). Conversely, recent reports suggest that diabetic state promotes PDAC and renders it highly aggressive, resistant to the existing therapies, and is associated with extremely poor prognosis (3, 4, 10–12). Hence, PDAC and diabetes exhibit a unique bidirectional relationship, with diabetes being both an effect and etiological factor of the pancreatic cancer (3, 4, 11).

Overexpression of heparanase (the only known mammalian endoglycosidase capable of degrading glycosaminoglycan heparan sulfate [HS]) is a characteristic feature of PDAC and correlates with its agressiveness/poor prognosis (13–17). HS proteoglycans are ubiquitously found both at the cell surface and in the extracellular matrix (ECM) (18–20). HS chains bind to and assemble ECM proteins, thus playing important roles in ECM integrity and cell-ECM interactions (18–20). In addition, HS chains regulate the activity of a variety of bioactive molecules (i.e., cytokines, growth factors) at the cell surface and in the ECM (21–24). The link between heparanase and PDAC progression is well-established (14–17) and the underlying molecular/cellular mechanisms include increased invasiveness (13, 25) and creation of tumor-promoting inflammatory environment (26). However, given the lack of heparanase expression in normal pancreatic tissue (13, 27), the regulatory mechanism(s) responsible for induction of the enzyme in PDAC are largely unknown.

Notably, heparanase was implicated in diabetes and its complications (17, 28–32). Moreover, previous research revealed molecular mechanism responsible for heparanase induction in immune, endothelial, and epithelial cells by several diabetic milieu components, i.e., high glucose, advanced glycation end-products, free fatty acids (29–31, 33–36). These findings, along with the impaired glucose metabolism that typically occurs in PDAC patients (3, 4), prompted us to hypothesize that in the setting of pancreatic carcinoma and associated hyperglycemia, constituent(s) of the diabetic milieu could be responsible for heparanase induction in PDAC cells.

Here, applying *in vivo* model of diet-induced metabolic syndrome [a cluster of conditions that includes hyperglycemia, insulin resistance, hyperinsulinemia, diabetes, and obesity (37)], we found that accelerated PDAC progression in mice with impaired glucose metabolism coincided with induction of heparanase in pancreatic tumors. *In vitro*, we demonstrated that advanced glycation end-products [AGE, oxidative derivatives resulting from hyperglycemia, whose levels are increased in clinical/experimental diabetes (38–42)] and its receptor (RAGE) are responsible for upregulation of heparanase in PDAC cells. AGEs form at a constant but slow rate in the normal body, however, their formation is markedly accelerated in diabetes because of the increased availability of glucose. Given deterioration in glycemic control in a majority of PDAC patients (2–4), these findings provide molecular explanation for induction of heparanase in pancreatic carcinoma. Moreover, taken together with the previously demonstrated causal role of the enzyme in PDAC progression (13, 26), our observations indicate that heparanase may be a part of the bi-directional link between diabetes and pancreatic tumorigenesis.

## Materials and Methods

### Cell Culture

The mouse pancreatic carcinoma cell line Panc02 [(43), a generous gift from M. Dauer (Munich, Germany)], and human pancreatic carcinoma cell lines MIA PaCa2 and PANC-1 (authenticated by STR profiling at the Genomics Center of the Biomedical Core Facility, Technion University, Israel), was grown in DMEM supplemented with 1 mM glutamine, 50 μg/ml streptomycin, 50 U/ml penicillin and 10% FCS (Biological Industries) at 37°C and 8% CO_2_. At 60–80% confluence, cells were maintained for 24 h in serum-free DMEM, and either remained untreated or were incubated with AGE (AGE-BSA, catalog #JM-2221–10; MBL International Corporation), or BSA (Sigma-Aldrich). In some experiments Panc02 cells were pretreated with RAGE neutralizing antibody (AF1179, R&D Systems) or TAK-242, TLR4 inhibitor (InvivoGen). The final endotoxin levels in experimental media containing AGE/BSA were 0.024–8 pg/mL, which were significantly lower than the concentrations typically found in diabetic patients (44), or than those required to activate Toll-like receptor (TLR) 4 or the classic NFκB pathway (45, 46). Cells were lysed and processed for RNA isolation. In some experiments, cells were cultured on glass coverslips (12 mm; Carolina Biological Supply Company), fixed with 100% ice-cold methanol and processed for imunofluorescent staining.

### Mouse Model of Metabolic Syndrome and Concurrent Pancreatic Carcinoma

Nine week-old male C57BL/6J mice (*n* = 10 per experimental group) were fed for 14 consecutive weeks with either regular (control) diet [Teklad 2018S] or the diabetogenic high fat diet (Teklad TD.06414), as in Montgomery et al. (47), Pettersson et al. (48), and Sandu et al. (49). At week 12, when experimental mice developed metabolic syndrome and became hyperglycemic, Panc02 pancreatic carcinoma cells were injected subcutaneously (10^6^ cells per mouse). Volume of tumors was monitored for 2 weeks following injection, then animals were sacrificed and tumors were snap-frozen for protein extraction. Part of the tumor tissue was processed for histology. Mice were kept under pathogen-free conditions; all experiments were performed in accordance with the Hebrew University Institutional Animal Care and Use Committee.

### Antibodies

Immunoblot analysis and immunostaining were carried out with the following antibodies: anti-phospho-AKT Ser 473 (Cell Signaling), anti-phospho NFκB p65 Ser276 (Cell Signaling Technology); anti-actin (Abcam); and anti-heparanase monoclonal antibody 01385–126, recognizing both the 50-kDa subunit and the 65-kDa proheparanase (50), which was provided by Dr. P. Kussie (ImClone Systems).

### Immunoblotting

Tumor tissue samples were homogenized in lysis buffer containing 0.6 % SDS, 10 mM Tris-HCl, pH 7.5, supplemented with a mixture of protease inhibitors (Roche), and phosphatase inhibitors (Thermo Scientific). Equal protein aliquots were subjected to SDS-PAGE (10% acrylamide) under reducing conditions, and proteins were transferred to a polyvinylidene difluoride membrane (Millipore). Membranes were blocked with 3% BSA for 1 h at room temperature and probed with the appropriate antibody, followed by horseradish peroxidase–conjugated secondary antibody (KPL) and a chemiluminescent substrate (Biological Industries). Band intensity was quantified by densitometry analysis using Scion Image software.

### Immunohistochemistry

Paraffin-embedded slides were deparaffinized and incubated in 3% H_2_O_2_. Antigen unmasking was carried out by heating (20 min) in a microwave oven in 10 mmol/L Tris buffer containing 1 mmol/L EDTA. Slides were incubated with primary antibodies diluted in CAS-Block (Invitrogen) or with CAS-Block alone, as a control. Appropriate secondary antibodies (Nichirei) were then added, and slides were incubated at room temperature for 30 min. Mouse stain kit (Nichirei) was used when primary mouse antibodies were applied to stain mouse tissues. Color was developed using the DAB Substrate Kit (Thermo Scientific) or Zymed AEC Substrate Kit (Zymed Laboratories), followed by counterstaining with Mayer's Hematoxylin. Controls without addition of primary antibody showed low or no background staining in all cases. Immunohistochemistry was scored based on staining intensity, as described in figure legends.

### Immunofluorescence

For immunofluorescence analysis, DyLight 549 donkey anti-mouse and Cy™3 donkey anti-rabbit (The Jackson Laboratory) antibodies were used as secondary antibodies. Nuclear staining was performed with 1,5-bis{[2-(di-methylamino)ethyl]amino}-4,8-dihydroxyanthracene-9,10-dione (DRAQ5) (Cell Signaling). Images were captured using a Zeiss LSM 5 confocal microscope and analyzed with Zen software (Carl Zeiss) and ImageJ software.

### Analysis of Gene Expression by Quantitative Real Time PCR (qRT-PCR)

Total RNA was isolated from 3 x 10^6^ cells using TRIzol (Invitrogen), according to the manufacturer's instructions, and quantified by spectrophotometry. After oligo (dT)-primed reverse transcription of 1 μg of total RNA, the resulting cDNA was amplified using the primers listed below. Real-time quantitative PCR (qRT-PCR) analysis was performed with an automated rotor gene system RG-3000A (Corbett Research). The PCR reaction mix (20 μl) was composed of 10 μl QPCR sybr master mix (Finnzymes), 5 μl of diluted cDNA (each sample in triplicate) and a final concentration of 0.3 μM of each primer. Hypoxanthine guanine phosphoribosyl transferase (HPRT) primers were used as an internal standard. The following primers were utilized: human HPRT sense: 5′-GCTATAAATTCTTTGCTGACCTGCT-3′, antisense: 5′-ATTACTTTTATGTCCCCTGTTGACTG-3′; human heparanase sense: 5′- GTTCTAATGCTCAGTTGCTCCT−3′, antisense: 5′-ACTGCGACCCATTGATGAAA-3′; mouse HPRT sense: 5′-GTC GTG ATT AGC GAT GAA-3′, antisense: 5′-CTC CCA TCT CCT TCA TGA CAT C-3′; mouse heparanase sense: 5′-ACT TGA AGG TAC CGC CTC CG-3′, antisense: 5′-GAA GCT CTG GAA CTC GGC AA-3′; mouse COX-2 sense: 5′-GGG TGT CCC TTC ACT TCT TTC A-3′, antisense: 5′-TGG GAG GCA CTT GCA TTG A-3′; mouse IL-6 sense: 5′- AGC CAG AGT CCT TCA GAG AGA TAC-3′, antisense: 5′- GCC ACT CCT TCT GTG ACT CC-3′, mouse TNF-α, sense: 5′-CAT CTT CTC AAA ATT CGA GTG ACA-3′, antisense: 5′-TGG GAG TAG ACA AGG TAC AAC CC-3′.

### Statistical Analysis

The results are presented as the mean ±SD or ±SE. *P* ≤ 0.05 were considered statistically significant. Statistical analysis was performed using unpaired Student's *t-*test. All statistical tests were two-sided.

## Results

### Dysregulation of Glucose Metabolism Accelerates PDAC Progression and Induces Heparanase Expression

**Figure 1:**
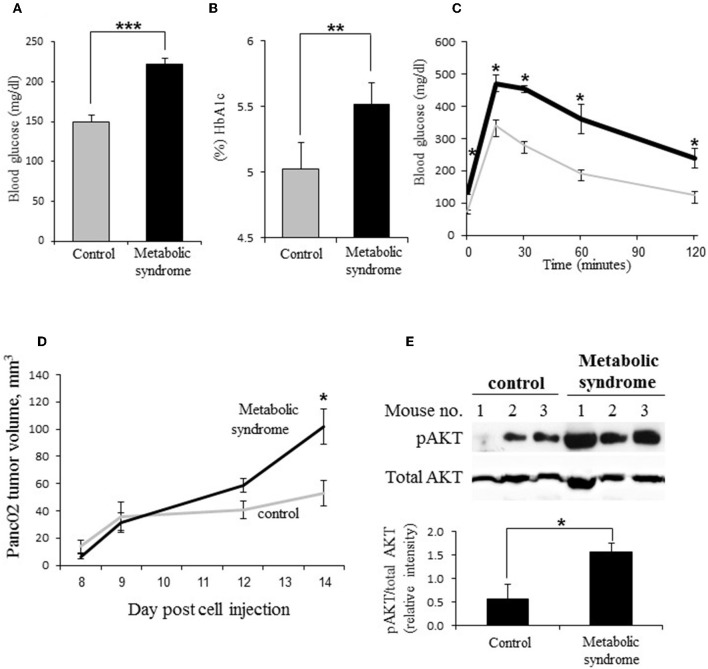
Dysregulation of glucose metabolism accelerates PDAC progression *in vivo*. **(A–C)** Impairment of glucose metabolism in male C57BL/6J mice with diet-induced metabolic syndrome. **(A)** Blood glucose levels [mg/dL], **(B)** glycated hemoglobin [HbA1c, %], and **(C)** glucose tolerance (determined by the i.p. glucose tolerance test [GTT]) in male C57/Bl6 mice following 12 weeks of diabetogenic high fat diet (**A,B**: black bars; **C**: black line) or regular (control) diet (**A,B**: gray bars; **C**: gray line). Error bars represent ±SD. Two-sided Student's *t*-test *p ≤ 0.003, **p = 0.013, ***p = 0.0002; *n* ≥ 5 mice per condition. **(D,E)** On week 12, mouse pancreatic carcinoma Panc02 cells were injected subcutaneously (10^6^ cells per mouse). **(D)** Volume of Panc02 tumors grown in mice with diet-induced metabolic syndrome (black line) and control mice (gray line) was monitored for 14 days. Error bars represent ±SE. Two-sided Student's *t*-test *p < 0.02. **(E)** Top: Increased levels of phospho-AKT (pAKT) in Panc02 tumors of mice with metabolic syndrome as compared to control mice. Bottom: The band intensity was quantified using ImageJ software; intensity ratio for pAKT/total AKT is shown, error bars represent ±SE. Two-sided Student's *t*-test *p = 0.048; *n* ≥ 3 mice per condition.

Deterioration in glycemic control is characteristic of PDAC: hyperglycemia has been repeatedly observed in majority (according to some reports—up to 85%) of PDAC patients (2–4); and epidemiologic studies report increased incidence of pancreatic carcinoma in diabetic populations (6, 7). Thus, to investigate heparanase regulation in PDAC under dysregulated glucose metabolism, we utilized a murine experimental system, based on Panc02 mouse pancreatic carcinoma cells growing in C57BL/6J mice with the diet-induced metabolic syndrome, as described in Methods. Diet-induced metabolic syndrome in male C57BL/6J mice represents a reliable model, which closely parallels metabolic abnormalities in diabetic patients, such as increased circulating concentrations of glucose, hyperinsulinemia and impairment of glucose tolerance (47–49). Following 12 weeks of the diet intake, metabolic syndrome/impaired glucose metabolism was documented in experimental mice fed with high fat diet (but not in the control mice fed with the regular diet), as manifested by hyperglycemia, significantly increased glycated hemoglobin A1c (HbA1c) blood levels and glucose intolerance (Figures 1A–C), along with hyperinsulinemia (Supplementary Figure 1) and increased body weight. Then, both control (normoglycemic) and experimental (hyperglycemic) mice were injected subcutaneously with Panc02 cells, as described in Methods. In agreement with previous reports (51, 52), growth of Panc02 pancreatic carcinoma *in vivo* was markedly accelerated in hyperglycemic mice (Figure 1D). Additionally, tumors grown in hyperglycemic mice expressed markedly elevated levels of phospho-AKT [pAKT] (Figure 1E), one of the hallmarks of the PDAC tumorigenesis (53, 54).

**Figure 2:**
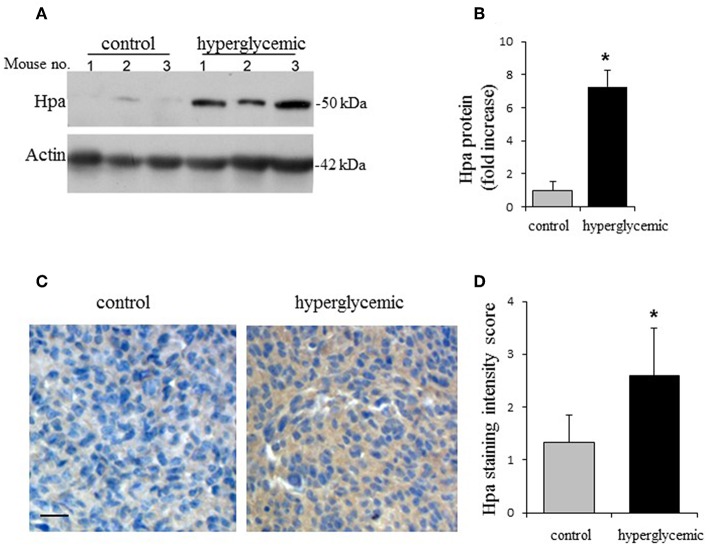
Hyperglycemic conditions induce expression of heparanase in Panc02 pancreatic carcinoma *in vivo*. **(A,B)** Heparanase protein (Hpa) levels in the Panc02 tumors derived from control (normoglycemic) and hypeglycemic mice. **(A)** Lysates of tumor tissue were analyzed by immunoblotting. **(B)** The band intensity was quantified using ImageJ software; intensity ratio for Hpa/actin is shown, error bars represent ±SD. Two-sided Student's *t*-test *p < 0.02; *n* ≥ 3 mice per condition. **(C)** Immunostaining (brown) of Panc02 tumor tissue sections with the anti-heparanase antibody diluted (1:200) in CAS-Block was performed as described in section Materials and Methods. Scale bar: 50 μm. **(D)** Sections were scored in a blinded fashion according to the heparanase staining intensity (low/no staining = 1; medium staining = 2; high staining = 3). *n* ≥ 5 mice per condition, at least 5 fields per tumor section were analyzed. The data shown are the mean ±SD of staining scores. Two-sided Student's *t*-test *p = 0.03.

We next compared heparanase expression in Panc02 tumors grown in experimental vs. control groups, applying immunoblotting. As shown in Figures 2A,B, markedly increased levels of heparanase protein were detected in Panc02 tumors growing in hyperglycemic, as compared to control (normoglycemic) mice. In agreement, quantitative RT-PCR analysis revealed ~2-fold increase in heparanase mRNA levels in hyperglycemic vs. control mice. Additionally, immunostaining of the mouse tumor tissues with heparanase antibody revealed that Panc02 carcinoma cells, rather than host-derived stromal cells, represent the main source of the enzyme in tumors growing in hyperglycemic mice (Figures 2C,D).

### AGE Induces Heparanase Expression in PDAC Cells *in vitro*

**Figure 3:**
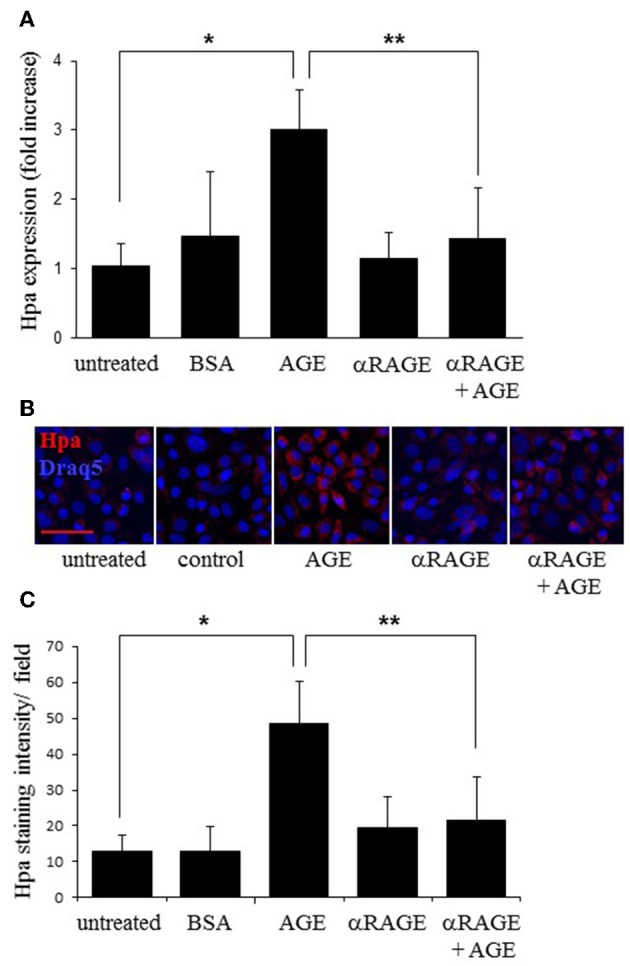
Heparanase upregulation *in vitro* is mediated by AGE/RAGE signaling. **(A,B)** Panc02 cells were either untreated, or incubated (24 h, 37°C) with 200 μg/mL of AGE or BSA (control), with or without pretreatment with RAGE neutralizing antibody (αRAGE, 12 μg/mL) and analyzed for heparanase (Hpa) mRNA expression by quantitative real time PCR **(A)** and immunostaining **(B)**. Error bars represent ±SD. Two-sided Student's *t*-test *p < 0.008, **p < 0.001. **(B)** Cell nuclei were counterstained with DRAQ5 (blue). Scale bar: 50 μm. **(C)** Heparanase staining intensity was quantified using ImageJ Software per microscopic field, based on at least 4 fields per condition. Data shown are the mean staining intensity per 0.03 mm^2^ microscopic field. Error bars represent ±SD. Two-sided Student's *t*-test *p < 0.002, **p < 0.004.

It was previously shown that various components of the diabetic milieu, including high glucose, free fatty acids, AGE, inflammatory cytokines (IL-6, TNF-α), upregulate heparanase in cells of non-pancreatic origin (29–31, 33–35, 50, 55). Importantly, increased levels of the aforementioned diabetic milieu constituents are present in the mouse model of metabolic syndrome/diabetes, used in our study (42, 47, 56–58). We therefore tested effects of various diabetic milieu components on heparanase expression in Panc02 cells *in vitro*. While treatment with either high glucose, fatty acids, insulin, or IL-6 failed to induce the enzyme expression (Supplementary Figure 2), treatment with AGE significantly increased expression of heparanase mRNA in Panc02 cells (Figure 3A). Immunofluorescent staining analysis also demonstrated increased heparanase protein levels in Panc02 cells following AGE treatment *in vitro* (Figures 3B,C), echoing *in vivo* observations (Figures 2C,D). Similar increase in heparanase expression in the presence of AGE was revealed in human PDAC cell lines MIA PaCa-2 and PANC-1 (Supplementary Figure 3).

AGE, whose formation is particularly augmented in diabetes due to combined effects of hyperglycemia and oxidative stress (39, 40), interact with the receptor for advanced glycation end products [RAGE], a multiligand receptor, expressed by numerous cell types, including PDAC cells (59, 60). Additionally, AGE are among the endogenous ligands known to activate toll-like receptor 4 (TLR4) (61–63), which is also expressed by PDAC cells (64, 65). As reported in Vaz and Andersson (64) and Kang et al. (65) and confirmed by qRT-PCR, Panc02 cells express both RAGE and TLR4. Responsiveness of Panc02 cells to AGE stimulation was further supported by upregulation of IL-6 and COX-2 following AGE treatment (Supplementary Figures 4A,B), as well as enhanced NFκB signaling, evidenced by increased levels/nuclear localization of phospho-p65 in AGE-treated Panc02 cells (Supplementary Figure 4C). Notably, it was previously shown that NFκB is involved in heparanase up-regulation in PDAC cells (66). Since NFκB activation is a known consequence of either TLR (67) or RAGE (68, 69) signaling, we next applied inhibitory approach to distinguish between these two pathways. While presence of TLR4-specific inhibitor TAK242 (70) did not affect heparanase induction by AGE in our system (Supplementary Figure 5), presence of RAGE-neutralizing antibody significantly decreased AGE-mediated heparanase induction, both at the mRNA and protein level (Figures 3A–C).

## Discussion

Among six cancer types attributable to diabetes (71), PDAC and diabetes display a unique reciprocal connection: PDAC is a presumed cause of derangement in glucose metabolism in a large number of cases, while diabetic state is known to promote pancreatic tumor progression (3, 4, 11, 71). Diabetes and PDAC are two heterogeneous diseases with a tremendous impact on health: PDAC has the lowest 5-year relative survival rate compared with all other solid tumor malignancies (1) and diabetes has become a pandemic (72). Thus, identification of pathways linking PDAC and impaired glucose metabolism is of high importance.

Here, applying mouse model of metabolic syndrome/diabetes and concurrent pancreatic carcinoma, we show that diabetic state leads to induction of heparanase expression in PDAC (Figures 1, 2). This induction appears to be driven by AGE (Figure 3), a well-characterized member of the diabetic milieu. It should be noted that the limitation of the present study is that the single model was used for *in vivo* confirmation—due to enormous complexity of both diseases (PDAC and diabetes) it remains extremely challenging to establish additional mouse models faithfully reflecting concurrent pancreatic tumor progression and diabetes.

Given abundant evidence implicating heparanase in PDAC pathogenesis/aggressiveness/therapy resistance (13–17, 25), our finding may provide a partial explanation for the mechanism through which diabetic state contributes to pancreatic carcinoma progression. Indeed, elevated levels of the enzyme have been found in PDAC tissue samples (13) and in body fluids of patients with active pancreatic cancer disease as compared to healthy donors (16). Pancreatic cancer patients whose tumors exhibit high levels of heparanase mRNA had a significantly shorter postoperative survival time than patients whose tumors contained relatively low levels (13, 15, 73). Heparanase is a highly significant independent variable for lymph node metastasis in pancreatic cancer patients, further supporting crucial involvement of the enzyme in PDAC progression (14). Importantly, the aforementioned epidemiological observations are backed by the experimental data demonstrating accelerated tumor growth/increased invasiveness in PDAC cells engineered to over-express heparanase (13, 15, 26), as well as a reduction of primary tumor progression/metastasis in murine models of PDAC following administration of heparanase-inhibiting compounds (25, 74). Although several mechanisms controlling expression of the enzyme in various tissues have been described (30, 31, 55, 75), regulation of heparanase induction in PDAC remained under investigated.

To promote PDAC development heparanase acts through augmented release of HS-bound growth factors, removal of extracellular barriers for invasion (13–16, 25) and creation of tumor-stimulating “aseptic” inflammatory conditions, i.e., increased production of IL-6 (a key cytokine driving pancreatic tumorigenesis) by heparanase-stimulated tumor associated macrophages (TAM) (26). In agreement with this mode of action, we found significantly increased levels of IL-6 (and in accordance—increased TAM infiltration) in heparanase-overexpressing Panc02 tumors derived from hyperglycemic mice (Supplementary Figure 6). Additionally, ability of the enzyme to augment insulin/insulin-like growth factor 1 receptor signaling (76, 77), along with the well-documented hyperinsulinemia in PDAC patients [either in the setting of new inset or long-standing diabetes (3, 8, 9)], suggests that in heparanase-rich PDAC microenvironment insulin is expected to induce stronger pro-tumorigenic response. Thus, heparanase induction appears to be a part of the mechanism(s) through which diabetic state promotes PDAC and renders it highly aggressive, therapy-resistant and associated with particularly poor prognosis (3, 4, 10–12).

On the other hand, emerging involvement of heparanase in diabetes, including its role in the islet/beta cell damage (32, 78–80), taken together with augmented production of the enzyme by pancreatic carcinoma cells under hyperglycemic conditions (this study), implies that the enzyme may exacerbate PDAC-associated diabetes. Indeed, pioneering studies by C R. Parish and his group identified multiple roles for heparanase in islet damage (originally—in the setting of type 1 diabetes) (32, 78–80). The islet-damaging heparanase actions include promotion of the leukocyte migration from pancreatic blood vessels and their passage across the islet basement membrane, as well as depletion of heparan sulfate which is required for beta cell survival (32, 78–80).

Importantly, beta cell damage, islet inflammation and islet-infiltrating leukocytes (particularly, macrophages) appear to promote type 2 diabetes (T2D) as well (81). Along with insulin resistance, beta cell dysfunction is a major component of T2D pathology, and clinical onset of T2D does not occur until beta cells fail to secrete sufficient insulin to maintain normoglycemia in the face of insulin resistance (81–85). Macrophages infiltrate islets in clinical and experimental T2D (86, 87) and are causally involved in beta cell dysfunction (81, 85, 88). Of note, patients with PDAC-associated diabetes often have high insulin levels and marked peripheral insulin resistance, similar to T2D [reviewed in (3)]. PDAC-associated diabetes also shares with T2D temporal relationship between insulin resistance, beta-cell dysfunction and development of impaired glucose tolerance (89): at earlier stages beta cells compensate for insulin resistance by increased insulin secretion, but progressive damage to beta cells leads to their dysfunction, deterioration in glycemic control, and at the later stage eventually leading to diabetes.

Given the secreted nature of the enzyme and its involvement in beta cell injury [via depletion of heparan sulfate (32, 78–80) and through tissue-damaging effects of the adversely-activated islet-infiltrating macrophages, similarly to those demonstrated in other pathologies (17, 26, 28, 50, 90)], it is conceivable that in hyperglycemic patients elevated levels of heparanase, originating from the tumor of exocrine pancreas (i.e., PDAC), can exert pathogenic effects within the endocrine compartment (i.e., islets), further impairing glucose metabolism and leading to the onset/aggravation of diabetes.

**Figure 4:**
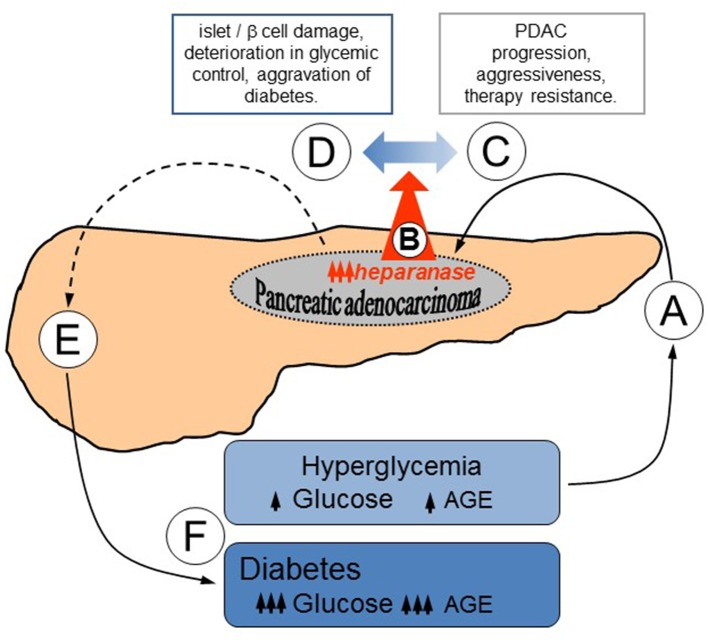
Proposed mode of heparanase action in sustaining bidirectional relationship between PDAC and diabetes. **(A)** Hyperglycemic state, which occurs in the majority of PDAC patients (2–4), results in heparanase overexpression in carcinoma cells via AGE-dependent mechanism **(B)**. **(C)** Elevated levels of heparanase promote PDAC development through several well-defined mechanisms, including augmented release of HS-bound growth factors, removal of extracellular barriers for invasion, and creation of tumor-stimulating inflammatory conditions (13, 15, 25, 26, 74). **(D)** In parallel, contribution of heparanase to the islet damage [originally described in the setting of type 1 diabetes (32, 78–80), but highly relevant to the pathogenesis of PDAC-associated diabetes as well] may impair beta cell function **(E)**, exacerbating diabetic state. **(F)** Aggravation of diabetes further escalates AGE production, advancing PDAC heparanase expression and its protumorigenic action.

Thus, our study not only reveals the mechanism of heparanase upregulation in PDAC, but also implies that the enzyme may contribute to a self-reinforcing sequence of events underlying bi-directional association between diabetes and PDAC (Figure 4): hyperglycemic state, that occurs in the majority of PDAC patients (Figure 4A), leads to heparanase overexpression in carcinoma cells via AGE-dependent mechanism (Figure 4B); increased levels of heparanase, in turn, promote PDAC progression (Figure 4C) through several previously-described mechanisms (13, 15, 25, 26, 74). In parallel, heparanase is capable of facilitating islet damage (Figure 4D), thus leading to beta cell dysfunction (Figure 4E), aggravating diabetic state and escalating AGE production, which further enhances PDAC heparanase expression and its protumorigenic action (Figure 4F).

Reciprocal relationships between PDAC and diabetes are certainly multifactorial in origin, and an array of molecular/cellular events underlying these relationships is far from being fully elucidated. Yet, our findings help to recognize the multilevel control that heparanase provides to heterotypic interactions among exocrine, endocrine and immune compartments of the pancreas in PDAC-diabetes link, suggesting that disruption of reciprocal causality between diabetes and PDAC through heparanase-targeting approaches may be of clinical benefit.

## Data Availability Statement

The datasets generated for this study are available on request to the corresponding author.

## Ethics Statement

The animal study was reviewed and approved by The Hebrew University Institutional Animal Care and Use Committee, Hebrew University of Jerusalem, Israel.

## Author Contributions

RG, EH, AR, AA, and DN conducted the experiments. AM and AG acquired the data. RG, AM, EH, and ME analyzed the data. TP and ME designed the studies. AM, AG, and TP reviewed the manuscript. ME was responsible for conceptualization, research design, supervised the study, and wrote the manuscript.

### Conflict of Interest

The authors declare that the research was conducted in the absence of any commercial or financial relationships that could be construed as a potential conflict of interest.
