# Supplementary material for: Regulation of Heparanase in Diabetes-Associated Pancreatic Carcinoma
Source: Front Oncol. 2019 Dec 10;9:1405. doi: 10.3389/fonc.2019.01405 (PMC6914686; doi:10.3389/fonc.2019.01405)
Supplement: Supplementary file 1 [file Data_Sheet_1.pdf]

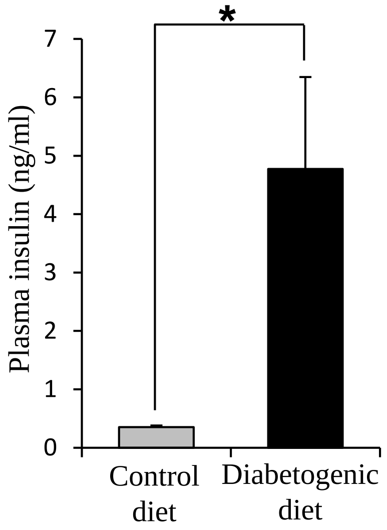

Supplementary figure 1. Plasma insulin concentrations detected in male C57/Bl6 mice following 12 weeks of diabetogenic diet (black bar) and in control mice fed with standard diet (grey bar). Error bars represent ±SD. Two-sided Student's t test * p=0.018, n≥5 mice per condition. For plasma insulin determination blood samples were collected through the caudal vein after 16 h of fasting. Insulin levels were measured using an enzyme-linked immunosorbent assay (Mercodia).

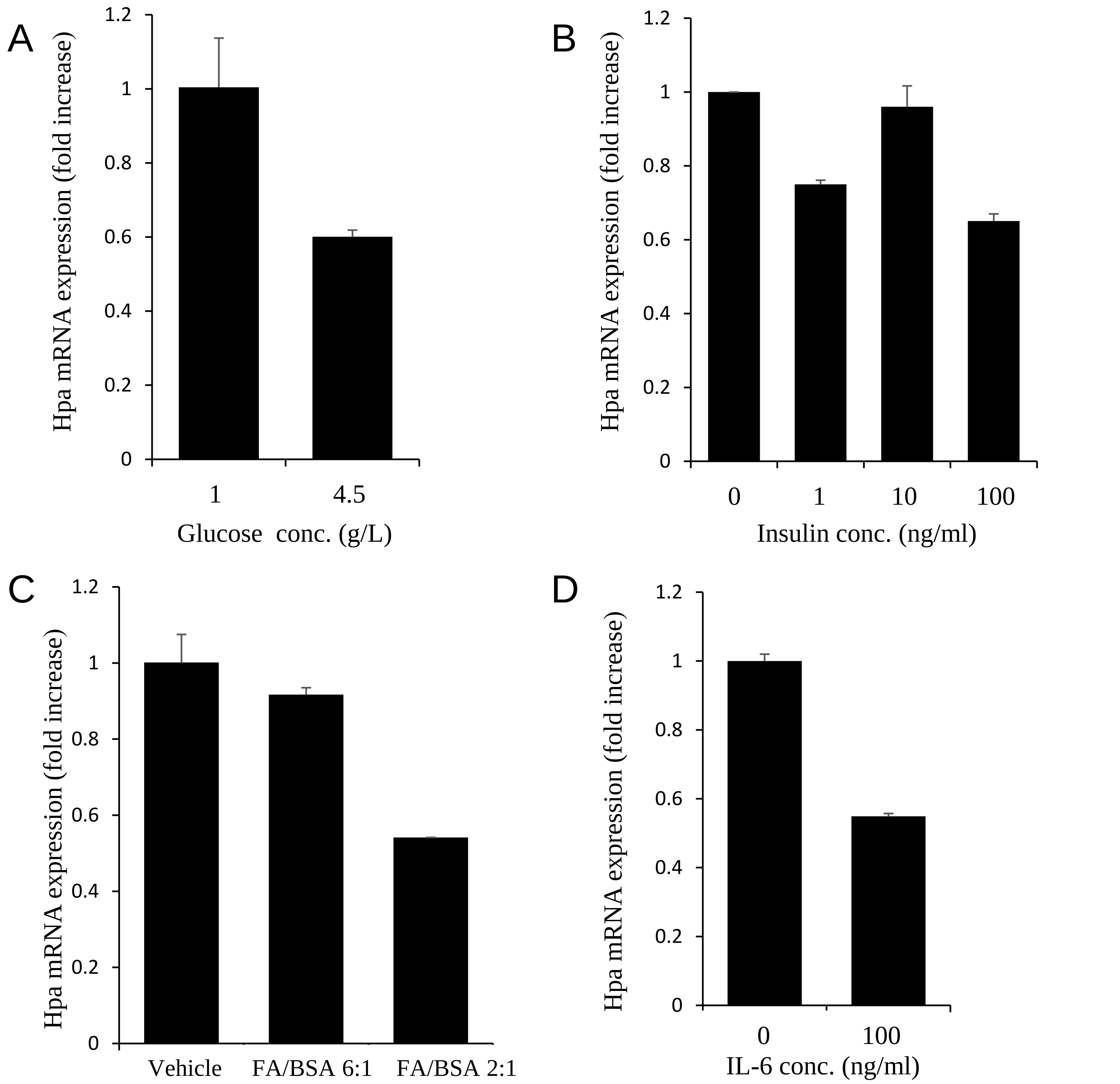

Supplementary Figure 2. Panc02 cells were incubated with several components of the diabetic milieu (as detailed below) and analyzed for heparanase (Hpa) mRNA expression by quantitative real time PCR. **A.** The cells were plated in medium containing either low (1.0 g/L) or high (4.5 g/L) glucose. Hpa expression was evaluated after 24 h. **B.** The cells were incubated (24 h, 37°C) in the absence or presence of insulin, at the indicated concentrations. Hpa expression was then evaluated. **C.** The cells were incubated (24 h, 37°C) with either BSA alone (vehicle) or with the palmitic fatty acid (FA, 200 μM, FA/BSA ratio 6:1 or 2:1). Hpa expression was then evaluated**. D.** The cells were incubated (24 h, 37°C) in the absence or presence of IL-6 (100 ng/ml). Hpa expression was then evaluated. Error bars represent ±SD.

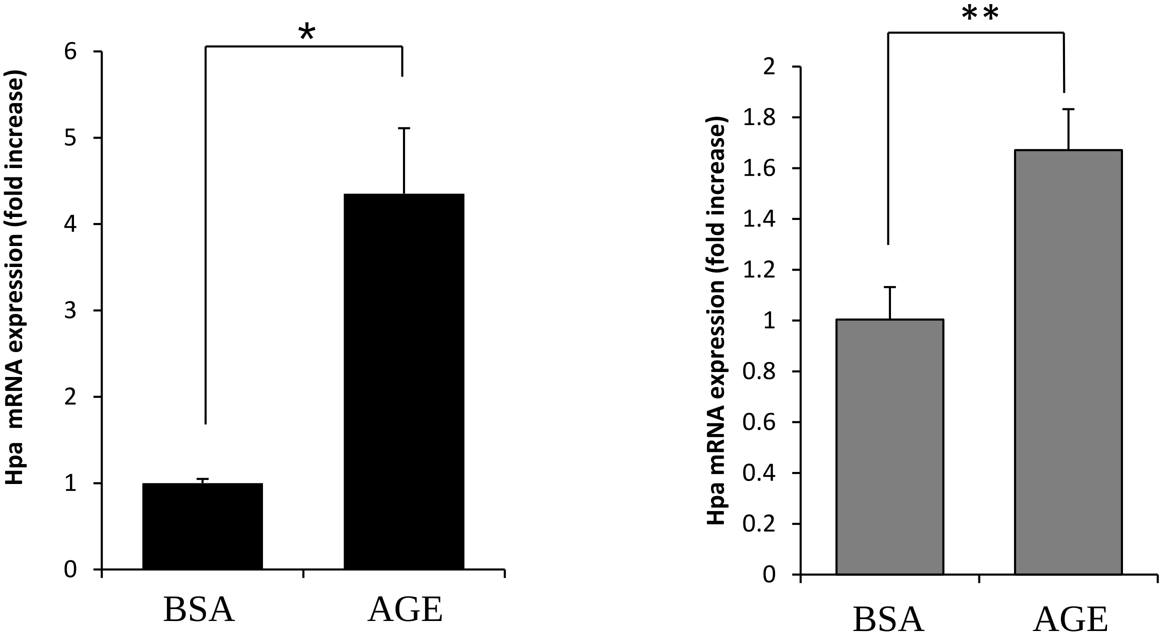

Supplementary Figure 3. Human PDAC cell lines MIA PaCa-2 (left panel) and PANC-1 (right panel) were incubated with 200 μg/mL of AGE or BSA (control) and analyzed for heparanase (Hpa) expression by quantitative real time PCR. Error bars represent ±SD. Two-sided Student's t test *p=0.0096, **p=0.017.

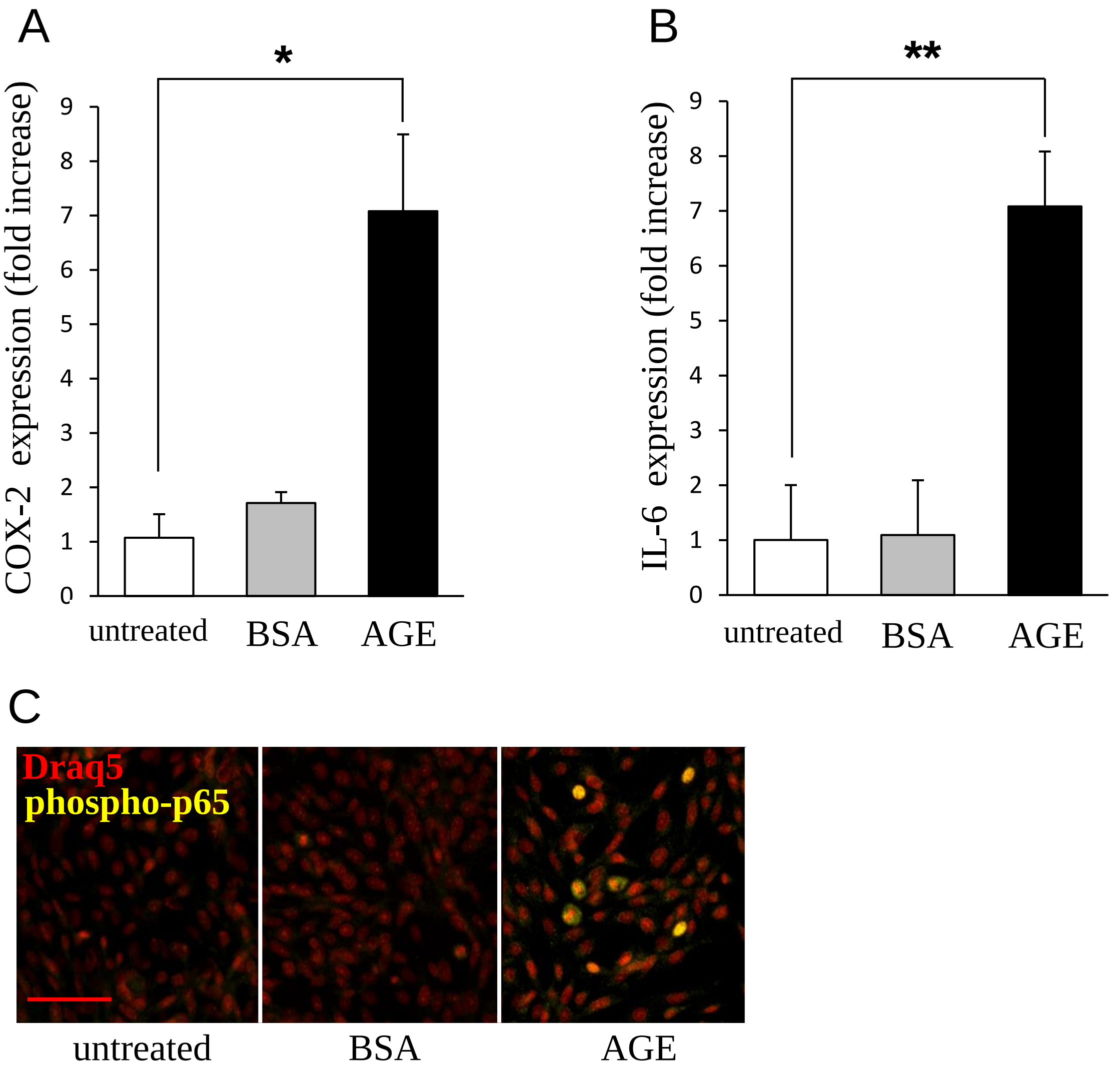

Supplementary Figure 4. Responsiveness of Panc02 cells to AGE treatment. Panc02 were either untreated, or incubated (24 h, 37°C) with AGE or BSA (200 µg/mL). AGE treatment upregulated expression of AGE-inducible molecules COX-2 (**A**) and IL-6 (**B**) as assessed by qRT-PCR. Error bars represent ±SD. Two-sided Student's t test* P<0.003, ** P<0.0002. **C**. Induction of NFκB signaling in Panc02, activated by AGE or BSA, was assessed by immunofluorescent analysis using anti-phospho-p65 (yellow) antibody. Cell nuclei were counterstained with DRAQ5 (red). Scale bar: 50µm. Note enhanced NFκB signaling in AGE-treated Panc02 cells, evidenced by increased levels/nuclear localization of phospho-p65. NFκB is normally sequestered in the cytoplasm by means of association with an inhibitory protein IκBα. Activation of NFκB involves stimulation of the IKK complex which triggers IκBα degradation and translocation of the active (phosphorylated) NFκB subunit p65 into the nucleus.

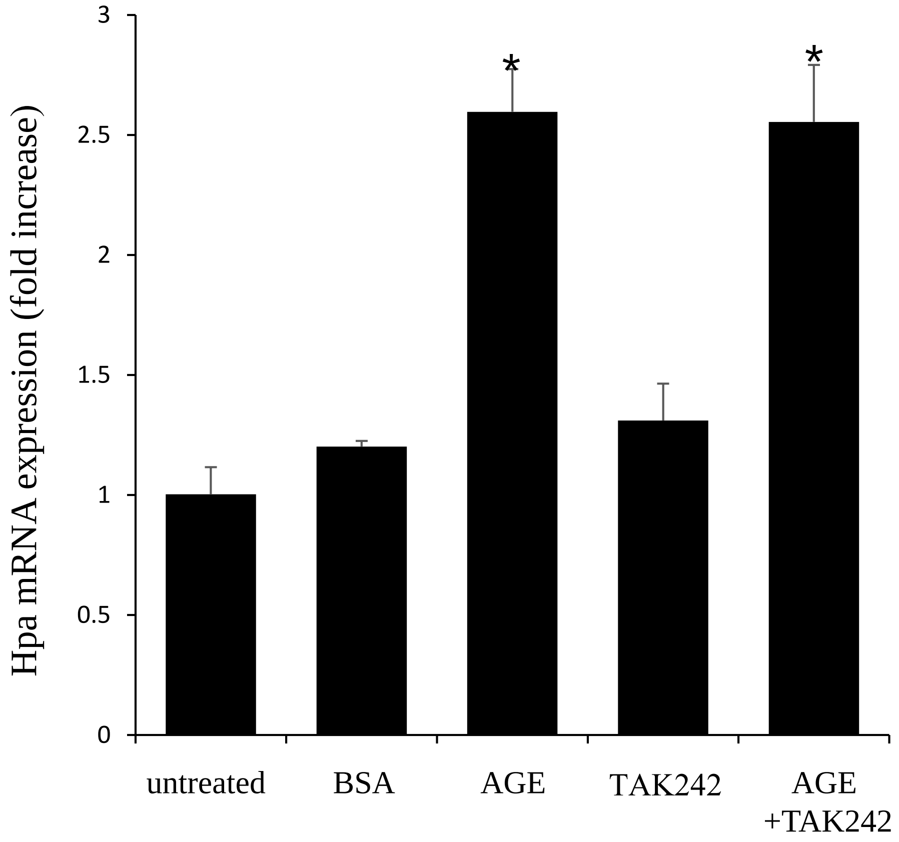

Supplementary Figure 5. Heparanase upregulation by AGE is not affected by the specific TLR4 inhibitor. Panc02 cells were either untreated, or incubated (24 h, 37°C) with 200 µg/mL of BSA (control) or AGE, with or without pretreatment with TLR4-specific inhibitor TAK-242 (1 µM), and analyzed for heparanase (Hpa) mRNA expression by quantitative real time PCR. Error bars represent ±SD. Two-sided Student’s t test *p<0.009.

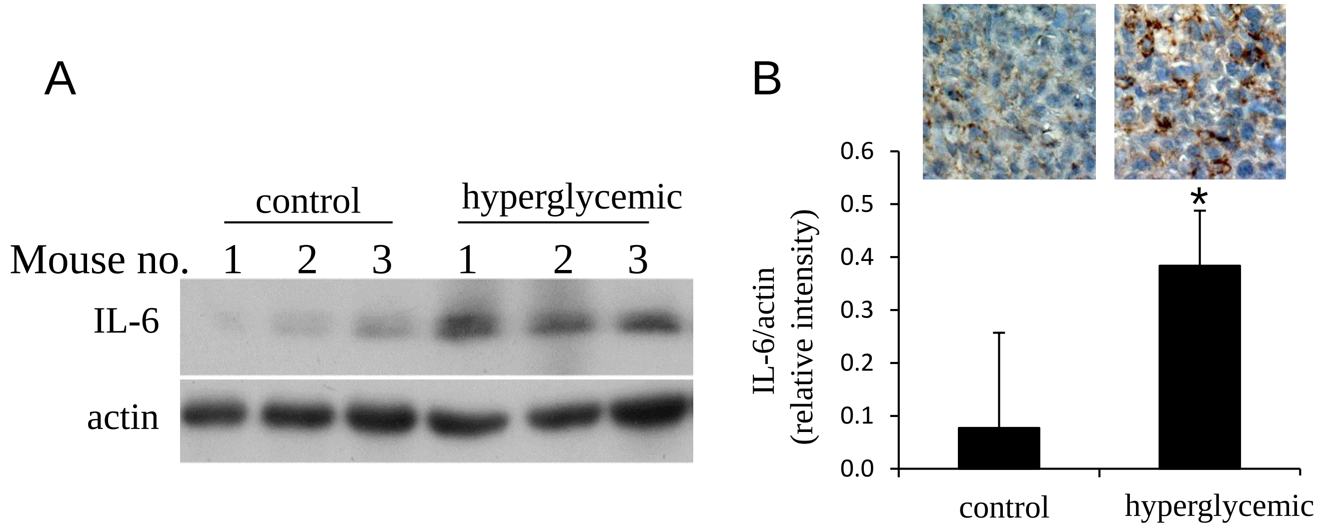

Supplementary figure 6. Augmented levels of IL-6 in heparanase-overexpressing Panc02 tumors derived from hypeglycemic mice. **A**. Lysates of tumor tissue were analyzed by immunoblotting. **B**. The band intensity was quantified using ImageJ software; intensity ratio for IL-6/actin is shown, error bars represent ±SD. Two-sided Student's t test *$p<0.03$; $n\geq3$ mice per condition. **Inset**. Immunostaining (brown) of Panc02 tumor tissue sections with F4/80 antibody (macrophage specific marker) reveals abundant infiltrating of the tumor tissue by macrophages.
